# CT and MRI radiomics in cardiovascular risk prediction: a systematic review and meta-analysis by the EuSoMII Radiomics Auditing Group

**DOI:** 10.1007/s00330-025-12236-2

**Published:** 2025-12-24

**Authors:** Armando Ugo Cavallo, Andrea Ponsiglione, Bernardo Pereira, Carlo Di Donna, Emmanouil Koltsakis, Federica Vernuccio, Mario Laudazi, Roberto Cannella, Salvatore Claudio Fanni, Tugba Akinci D’Antonoli, Renato Cuocolo

**Affiliations:** 1 Division of Radiology, Istituto Dermopatico dell’Immacolata (IDI), IRCCS, Rome, Italy; 2 Department of Advanced Biomedical Sciences, University of Naples Federico II, Naples, Italy; 3 Quantitative Imaging Biomarkers in Medicine, Quibim SL, Valencia, Spain; 4 Division of Radiology, Sant’Andrea University Hospital, Rome, Italy; 5 Department of Nuclear Medicine, Karolinska University Hospital Huddinge, Stockholm, Sweden; 6 Department of Clinical Science, Intervention and Technology (CLINTEC), Karolinska Institute, Stockholm, Sweden; 7 Department of Biomedicine, Neuroscience and Advanced Diagnostics (BiND), University of Palermo, Palermo, Italy; 8 Division of Radiology, Policlinico Tor Vergata, Rome, Italy; 9 Department of Translational Research, Academic Radiology, University of Pisa, Pisa, Italy; 10 Department of Pediatric Radiology, University Children’s Hospital Basel, Basel, Switzerland; 11 Department of Diagnostic and Interventional Neuroradiology, University Hospital Basel, Basel, Switzerland; 12 Department of Medicine, Surgery, and Dentistry, University of Salerno, Baronissi, Italy

**Keywords:** Cardiovascular system, Radiomics, Magnetic resonance imaging, Computed tomography, Meta-analysis

## Abstract

**Objectives:**

To conduct a comprehensive systematic review of the studies applying radiomics to CT and MRI for the evaluation of cardiac disease, and to perform a meta-analysis of their diagnostic accuracy, focused on cardiovascular events prediction. A secondary aim was to assess the methodological quality of cardiac imaging radiomics studies using the METRICS score.

**Materials and methods:**

Four investigators searched multiple medical literature archives (Scopus, Web of Science, and PubMed). The search was conducted from February 7th, 2021, to March 10th, 2025. Papers were also screened to identify studies for the prediction of cardiovascular events, defined as the occurrence of major cardiovascular events or myocardial ischemia. Methodological quality was assessed by using the METRICS tool. Diagnostic accuracy was estimated with pooled area under the curve (AUC).

**Results:**

A total of 202 studies were included in the final analysis. Seventeen papers were identified for the meta-analysis, of which 9 were considered eligible for analysis. 111 papers (55%) had CT as the imaging modality, and 91 (45%) papers had MRI. Overall, the average METRICS total score was 54.52% ± 15.89%. Meta-analysis showed pooled AUC of 0.81 (95% CI: 0.75–0.87), with a high level of heterogeneity (I² = 83.4%, τ² = 0.0068). Egger’s test for funnel plot asymmetry was statistically significant (*z* = −2.39, *p* = 0.017), suggesting potential publication bias.

**Conclusion:**

Radiomics in cardiac imaging holds potential, showing moderate quality and relatively high cumulative performance for the prediction of cardiovascular events.

**Key Points:**

***Question*** *What is the current methodological quality and pooled diagnostic performance of cardiovascular radiomics for predicting clinical events, based on a meta-analysis?*

***Findings*** *The average METRICS quality score was 54.52%. A meta-analysis showed a pooled AUC of 0.81 for event prediction, but with high heterogeneity and publication bias.*

***Clinical relevance*** *Assessing radiomics research methodological quality is crucial to enhance reproducibility and clinical applicability of radiomics pipelines. The evaluation of cumulative evidence for cardiovascular events prediction may guide clinical translation and future study design.*

**Graphical Abstract:**

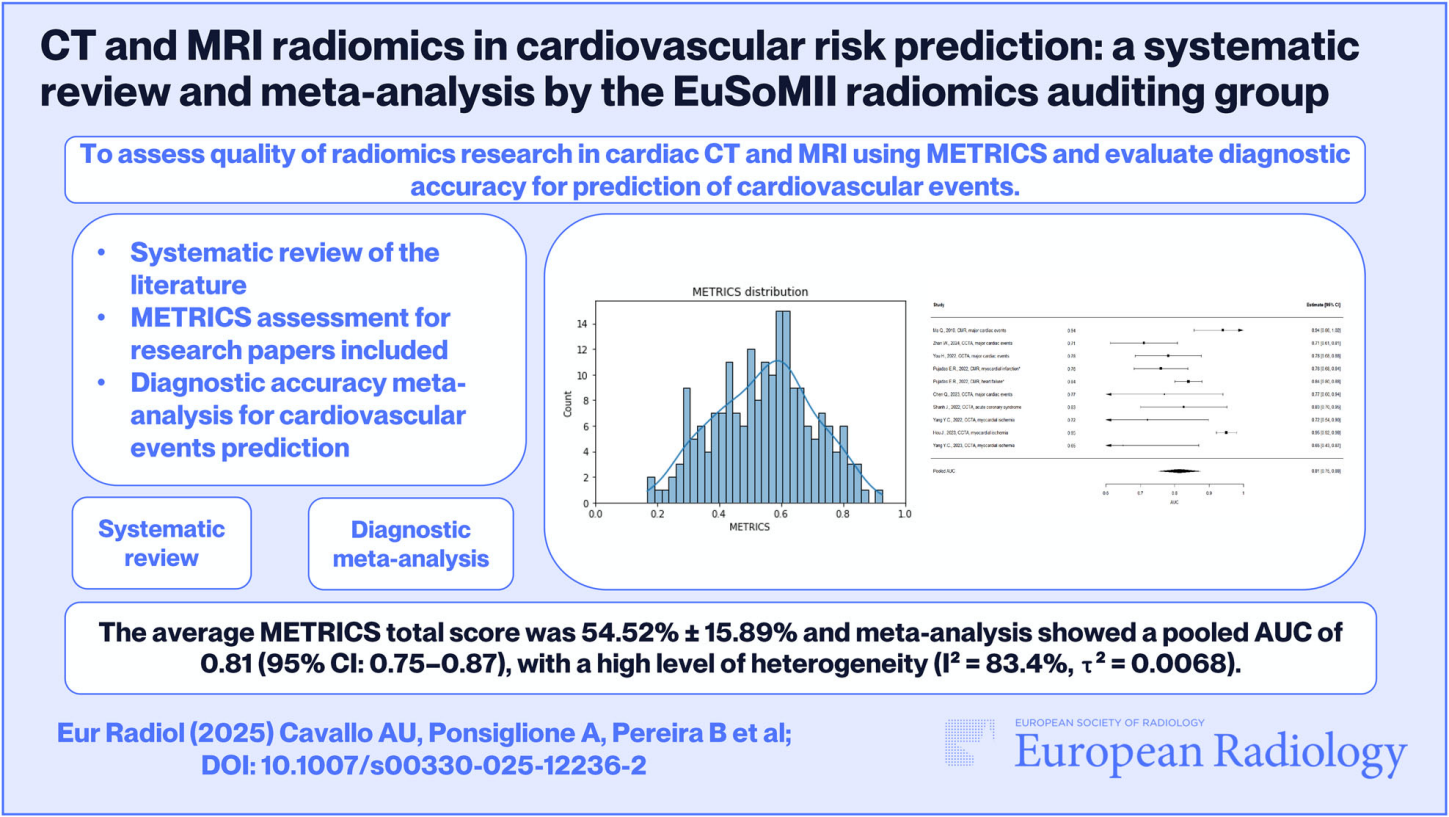

## Introduction

Radiomics encompasses a collection of applications based on the extraction of mineable feature data from biomedical images, which can reveal subtle patterns and characteristics not readily apparent to the human eye [1]. These novel biomarkers have shown great potential in training and improving artificial intelligence models, with an exponential growth both in terms of research output and use in medical devices [2, 3].

Originally pioneered in oncology, radiomics has increasingly been applied in cardiovascular imaging, where high-throughput feature extraction from CT and MRI scans can support the diagnosis and prediction of various myocardial and cardiovascular diseases [4, 5]. Despite its great potential, radiomics research in cardiovascular disease still suffers from major limitations related to heterogeneity in methodology [6].

In 2017, the radiomics quality score (RQS) was proposed [7], with the purpose of increasing the quality of radiomics research. In 2021, Ponsiglione et al conducted a systematic review of radiomics studies in cardiac CT and MRI, using RQS for the evaluation of research quality. Notably, they reported a median RQS of only 7, corresponding to a percentage score of 19.4% across 53 papers included in the analysis [8]. These results indicate that studies generally exhibited low methodological quality, thereby hindering the adoption of radiomics workflows into clinical practice.

In 2024, the European Society of Medical Imaging Informatics (EuSoMII) introduced the METhodological RadiomICs Score (METRICS) [9], a novel tool designed to systematically assess the methodological quality of radiomics studies. Developed through a modified Delphi process involving a large international panel of experts, METRICS aims to promote higher standards and improve reproducibility in radiomics research. Currently, no systematic review evaluates the quality of radiomics research in cardiac imaging using the METRICS tool.

The aim of this study was to conduct a comprehensive systematic review of the studies applying radiomics to CT and MRI for the evaluation of cardiac disease, and to perform a meta-analysis of their diagnostic accuracy, focused on cardiovascular events prediction. A secondary aim was to assess the methodological quality of cardiac imaging radiomics studies using the METRICS score.

## Materials and methods

### Literature search

Following the search strategy of the previous systematic review conducted by Ponsiglione et al [8], to identify records of interest, multiple medical literature archives (Scopus, Web of Science, and PubMed) were interrogated using the following string:

*(“textural” OR “radiomics” OR “texture” OR “histogram”) AND (“cardiac” OR “heart”) AND (“computed tomography” OR “CT” or “magnetic resonance” OR “MRI” OR “MR”)*

The search was conducted from February 7th, 2021 (the end date of the previous systematic review), to March 10th, 2025. After the removal of duplicates, abstracts were screened to identify and remove non-original investigations (e.g., letters, editorials, reviews, etc.), studies not focused on the topic of interest, and papers published in languages other than English. Manuscripts were independently screened by four investigators (A.P., T.A.D., C.D.D., A.U.C.). Investigators involved in this task annotated exclusion criteria and assigned each manuscript to a topic: Cardiovascular risk, Coronary Artery Disease, Arrhythmias, Myocarditis, Cardiomyopathies, Thrombotic Disease, Valvular Disease, Tumors, and Technical.

### Data collection and study evaluation

English full texts of the selected records were retrieved and independently screened by eight readers after random assignment (A.U.C., B.P., E.K., F.V., C.D.D., R.C., M.L., S.C.F.); each paper was assessed by one reader only. All participants had prior experience (1–5 years) in radiomics-based research papers. The readers assessed each paper using the dedicated METRICS calculator (https://metricsscore.github.io/metrics/METRICS). An additional investigator (R.C.), who is among the principal developers of the METRICS score, was consulted to resolve any interpretative challenges.

METRICS score is expressed as a 0–100% value, as well as discrete categorization as: 0 ≤ score < 20%, “very low”; 20 ≤ score < 40%, “low”; 40 ≤ score < 60%, “moderate”; 60 ≤ score < 80%, “good”; and 80 ≤ score ≤ 100%, “excellent” [9].

For diagnostic meta-analysis, one reader (A.U.C.) screened manuscripts assigned to the “Cardiovascular risk” and “Coronary Artery Disease.” Manuscripts where a prediction analysis of cardiovascular events was performed were selected, and then data about AUC and confidence intervals or standard error were collected. Manuscripts where measures of uncertainty were not reported were excluded.

In manuscripts where a 95% confidence interval was reported instead of the standard error, the standard error was calculated using the following formula:

### (CI upper limit − CI lower limit) / (2 × 1.96)

#### Statistical analysis

Statistical analysis was performed with R (V 4.4.2) [10].

Descriptive statistics were calculated, with continuous variables presented as mean ± standard deviation and categorical variables as percentages.

The Shapiro–Wilk test was used to assess normal distribution.

A subgroup analysis was performed to evaluate whether the METRICS score varied significantly according to the study aim using the Kruskal–Wallis test.

Correlations were assessed using Spearman’s rank correlation coefficient (rho).

A *p*-value less than 0.05 was considered statistically significant.

A diagnostic meta-analysis was conducted using the random-effects model to synthesize diagnostic performance across multiple studies based on reported area under the curve (AUC) values. Each study provided an AUC estimate and its corresponding standard error (SE), from which variances were computed.

A random-effects model was fitted with restricted maximum likelihood estimation.

**Fig. 1:**
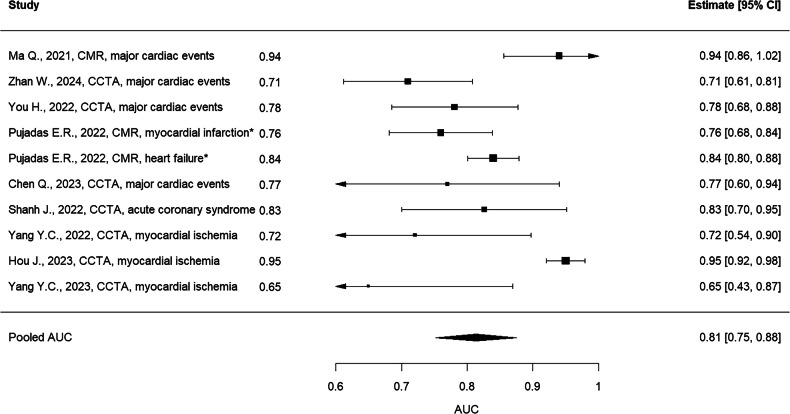
Forest plot of AUC (area under the curve) values from radiomics models in cardiac imaging

To visualize individual and pooled estimates, a forest plot was generated displaying the AUCs, 95% confidence intervals, and the overall summary effect (Fig. 1).

Predicted values and confidence intervals for the pooled AUC were calculated.

**Fig. 2:**
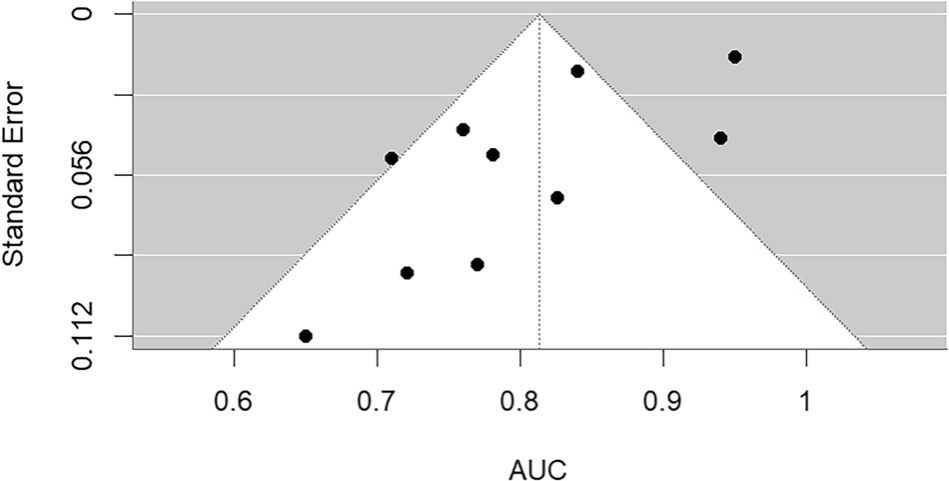
Funnel plot assessing publication bias in radiomics models for cardiac imaging: overall meta-analysis

Additionally, a funnel plot was used to assess potential publication bias, and Egger’s regression test was applied to formal tests for asymmetry (Fig. 2).

The trim-and-fill method was applied to estimate the number of potentially missing studies and to obtain a bias-adjusted pooled AUC.

Sensitivity analysis was performed using a leave-one-out approach, sequentially excluding each study and recalculating the pooled estimate. The observed variations were used to identify potentially influential studies and to assess the robustness of the meta-analytic results.

A univariable meta-regression was also conducted to explore the relationship between diagnostic performance (AUC) and study-level characteristics. Continuous variables (number of patients and METRICS score) were included according to data availability and clinical relevance. Random-effects models were used, and regression coefficients with 95% confidence intervals were reported.

Finally, a subgroup analysis was performed to assess whether the pooled AUC estimates differed according to key study-level features (prediction of myocardial ischemia or major adverse cardiovascular events (MACE); CT or MRI modality, myocardium or epicardial/pericoronary adipose tissue segmentation (EAT/PCAT)). Separate estimates for each subgroup were compared to explore sources of heterogeneity and potential effect modifiers.

The following packages were used: stat, metafor.

## Results

### Literature search

**Fig. 3:**
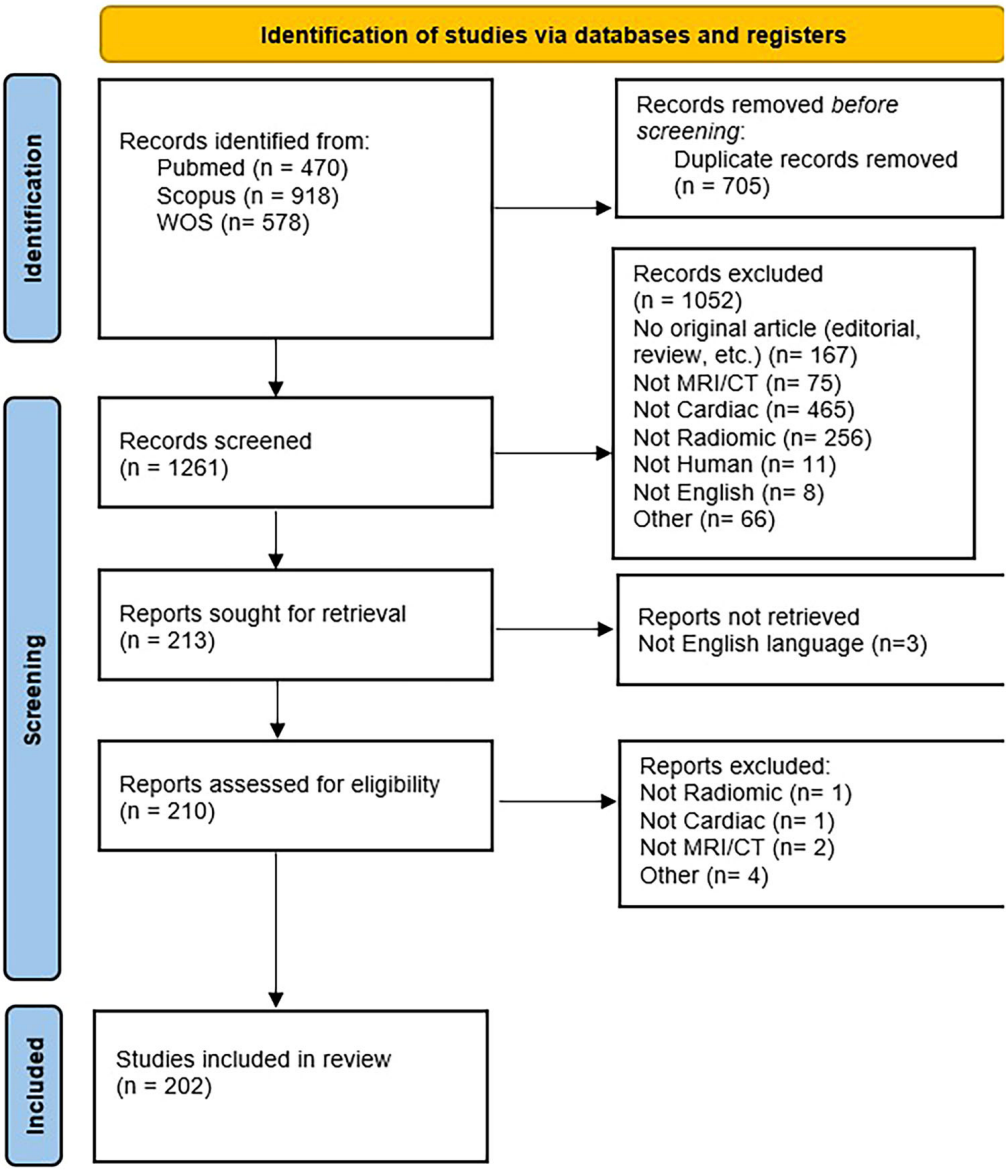
Prisma flowchart for literature search

The Prisma flowchart illustrating the steps of article identification, screening, and inclusion is reported in Fig. 3. In total, 1966 records were identified from the three repositories included in the search (PubMed, Scopus and Web of Science). After removing 705 duplicates and 1052 unrelated records, 213 studies were identified, of which 3 were excluded because they were not in the English language.

Thus, 210 records were assessed for eligibility, and 8 more were excluded for different reasons, as illustrated in Fig. 3. Finally, 202 manuscripts were available to be rated using METRICS.

### Data collection and study evaluation

Overall, 32 (15.84%) papers were published in 2021 (after February 7th till December 31st), 49 (24.26%) in 2022, 47 (23.27%) in 2023, 58 (28.71%) in 2024, and 16 (7.92%) in 2025 (January–March 10th). Regarding the imaging modality, 111 papers had CT as the imaging modality, and 91 papers had MRI.

**Fig. 4:**
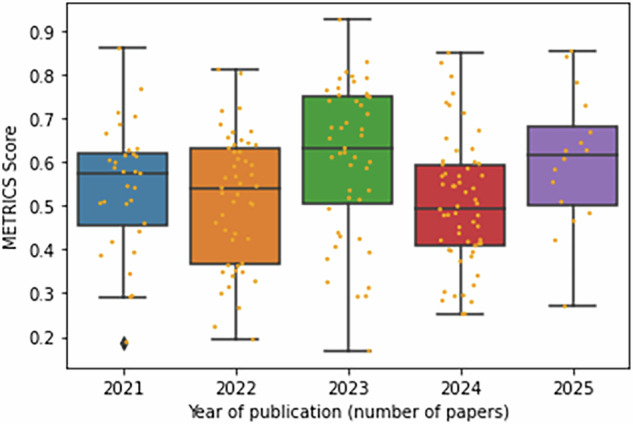
Boxplot and distribution of METRICS total score per year of publication

Overall, the average METRICs total score was 54.52% ± 15.89%, with 10 manuscripts (4.95%) rated as “Excellent,” 70 (34.65%) as “Good,” 81 (40.09%) as “Moderate,” 38 (18.81%) as “Low” and 3 (1.48%) as “Very Low.” The distribution of the total METRICS scores according to the publication year is illustrated in Fig. 4.

The correlation between METRICS scores and publication year was weak, with a Spearman coefficient (rho) of 0.04 (*p* = 0.96).

**Fig. 5:**
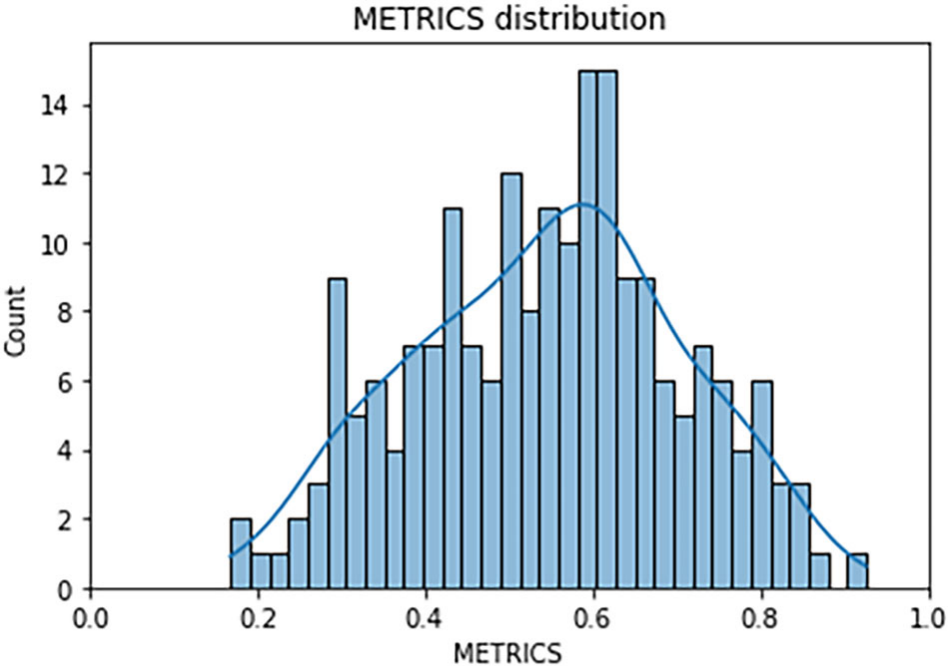
Histogram and kernel density of the total METRICS score in the analyzed papers

The Shapiro–Wilk test showed *p* = 0.16 for the overall METRICS score, indicating a normal distribution (Fig. 5).

Table 1 provides a summary of the METRICS items and conditions results.

**Table 1:** Summary of single items and conditions results, in absolute number and percentage

Items-Conditions	Number of “YES”	Percentage
Item#1: Adherence to radiomics and/or machine learning-specific checklists or guidelines	13	6.44%
Item#2: Eligibility criteria that describe a representative study population	173	85.64%
Item#3: High-quality reference standard with a clear definition	151	74.75%
Item#4: Multicenter	35	17.33%
Item#5: Clinical translatability of the imaging data source for radiomics analysis	142	70.30%
Item#6: Imaging protocol with acquisition parameters	166	82.18%
Item#7: The interval between the imaging used and the reference standard	127	62.87%
Condition#1: Does the study include segmentation?	195	96.53%
Condition#2: Does the study include fully automated segmentation?	46	22.77%
Item#8: Transparent description of segmentation methodology	134	66.34%
Item#9: Formal evaluation of fully automated segmentation*	18	39.13%
Item#10: Test set segmentation masks produced by a single reader or automated tool	101	50.00%
Condition#3: Does the study include hand-crafted feature extraction?	194	96.04%
Item#11: Appropriate use of image preprocessing techniques with transparent description	94	46.53%
Item#12: Use of standardized feature extraction software	141	69.80%
Item#13: Transparent reporting of feature extraction parameters, otherwise providing a default configuration statement	79	39.11%
Condition#4: Does the study include tabular data?	186	92.08%
Condition#5: Does the study include end-to-end deep learning?	11	5.45%
Item#14: Removal of non-robust features	119	58.91%
Item#15: Removal of redundant features	158	78.22%
Item#16: Appropriateness of dimensionality compared to data size	101	50.00%
Item#17: Robustness assessment of end-to-end deep learning pipelines**	6	54.55%
Item#18: Proper data partitioning process	125	61.88%
Item#19: Handling of confounding factors	47	23.27%
Item#20: Use of appropriate performance evaluation metrics for task	165	81.68%
Item#21: Consideration of uncertainty	163	80.69%
Item#22: Calibration assessment	44	21.78%
Item#23: Use of uni-parametric imaging or proof of its inferiority	166	82.18%
Item#24: Comparison with a non-radiomic approach or proof of added clinical value	111	54.95%
Item#25: Comparison with simple or classical statistical models	87	43.07%
Item#26: Internal testing	129	63.86%
Item#27: External testing	28	13.86%
Item#28: Data availability	36	17.82%
Item#29: Code availability	14	6.93%
Item#30: Model availability	5	2.48%

* % referred to Condition #2

** % referred to Condition #5

Supplementary Table S1 provides the radiomics features evaluated in the studies selected (when applicable).

### METRICS scores and category

Single groups’ scores are summarized in Table 2.

**Table 2:** METRICS score in different categories

	No. of papers	METRICS scores mean	SD	CT	MRI
Arrhythmias	11	52.51%	11.79%	8	3
Cardiomyopathies	51	56.22%	15.01%	7	44
Cardiovascular risk	31	59.02%	17.70%	22	9
Coronary artery disease	69	54.13%	15.60%	53	16
Myocarditis	3	46.23%	8.89%	1	2
Technical	23	47.34%	17.11%	9	14
Thrombotic disease	7	56.80%	20.54%	6	1
Tumors	2	56.30%	19.94%	1	1
Valvular disease	5	53.04%	8.75%	4	1

The Shapiro–Wilk test showed *p* > 0.05 for all categories except Tumors category, where the number of scores was too low for an adequate assessment (2 studies in total). For this reason, it was not possible to ensure the normal distribution of variables across all categories, and a non-parametric test was used for the comparison.

Comparison between different topics showed no statistically significant difference in METRICS scores (*p* = 0.43).

### Diagnostic meta-analysis

The diagnostic meta-analysis included studies that evaluated the use of radiomics for predicting cardiovascular risk or adverse cardiac outcomes, defined as the occurrence of MACE (e.g., cardiac death, heart failure, myocardial infarction, or cardiovascular hospitalization) or myocardial ischemia.

These outcomes were grouped together because they represent clinically relevant endpoints that can be consistently defined and measured across studies, allowing adequate standardization of methods and comparison of results within the meta-analysis. Papers were selected that relate to the use of radiomics in predicting the risk of cardiovascular events (such as sudden death, heart failure, or hospitalization due to cardiovascular events) as well as myocardial ischemia.

A total of 17 studies were selected; of them, 8 were excluded (3 because they did not report AUC analysis and 5 because they did not report confidence intervals or standard errors). Therefore, 9 studies were evaluated [11–19]. One paper [14] reported two separate analyses on myocardial infarction and heart failure that were considered separately.

A total of 10 studies were included in the overall meta-analysis evaluating diagnostic performance using AUC as the primary outcome, including 2967 patients in total were evaluated among studies (2035 for MACE and 932 for myocardial ischemia). Mean METRICS total score for selected papers was 63.7%. Table 3 summarizes the main characteristics of studies included in the diagnostic meta-analysis.

**Table 3:** Main characteristics of studies included in diagnostic meta-analysis

Study	AUC	SE	No. of patients	External validation	Multicenter	METRICS total score	Prospective	Data availability	Non-radiomics approach tested	Non-radiomics approach with higher AUC
Zhan W., 2024, CCTA, major cardiac events	0.71	0.05	239	No	No	38.40%	No	No	Yes	No
Chen Q., 2023, CCTA, major cardiac events	0.77	0.087	225 + 708 for external validation	Yes	Yes	80.60%	Yes	No	Yes	No
You H., 2022, CCTA, major cardiac events	0.781	0.049	288	No	No	75.20%	No	No	No	NA
Pujadas E.R., 2022, CMR, heart failure*	0.84	0.02	418	No	No	66.10%	No	No	Yes	No
Ma Q., 2021, CMR, major cardiac events	0.94	0.043	157	No	No	54.10%	No	No	No	NA
Shanh J., 2022, CCTA, acute coronary syndrome	0.826	0.064	180	No	No	65.60%	No	No	Yes	No
Yang Y.C., 2022, CCTA, myocardial ischemia	0.721	0.09	110	No	No	59.30%	No	No	No	NA
Pujadas E.R., 2022, CMR, myocardial infarction*	0.76	0.04	436	No	No	66.10%	No	No	Yes	No
Hou J., 2023, CCTA, myocardial ischemia	0.95	0.015	96	No	No	62.20%	No	No	Yes	No
Yang Y.C., 2023, CCTA, myocardial ischemia	0.65	0.112	90 + 20 (external validation)	No	Yes	71.70%	No	No	Yes	No

* The same paper included two studies with different populations

Supplementary Table S2 summarizes clinical features evaluated in selected manuscripts in diagnostic meta-analysis.

Five papers focused on the prediction of major cardiac events (hospitalization for heart failure, myocardial infarction, sudden cardiac death or heart failure), four of myocardial ischemia/acute coronary syndromes and one paper [14] analyzed two different populations: one for the prediction of myocardial ischemia and the other for the prediction of heart failure.

CT-based imaging was used in six studies, while MRI was the method of choice in four studies.

In six studies, the myocardium was segmented, whereas in the remaining four, segmentation focused on the EAT/PCAT.

Information on the software packages used in the included studies was also collected. None of the reported radiomics tools were CE- or FDA-marked for clinical use. Details of the analytical platforms employed are provided in Supplementary Table S3.

The pooled AUC was 0.81 (95% CI: 0.75–0.87), with a high level of heterogeneity (I² = 83.4%, τ² = 0.0068). The prediction interval ranged from 0.64 to 0.99, indicating substantial between-study variability. Egger’s test for funnel plot asymmetry was statistically significant (z = −2.39, *p* = 0.017), suggesting potential publication bias.

### Sensitivity analysis and trim and fill

Egger’s test indicated potential publication bias.

The trim-and-fill analysis imputed three missing studies on the right side of the funnel plot (Supplementary Fig. S1). After adjustment, the pooled AUC was 0.85 (95% CI: 0.79–0.91), with a prediction interval of 0.66–1.04. The bias-adjusted estimate was only slightly higher than the unadjusted value, suggesting that the influence of publication bias on the pooled diagnostic performance was modest, although substantial heterogeneity persisted (I² = 84.1%, *p* < 0.001).

The leave-one-out sensitivity analysis showed that the pooled AUC estimates ranged from 0.80 (95% CI: 0.74–0.86) to 0.83 (95% CI: 0.76–0.89) when each study was sequentially excluded (Supplementary Table S4).

No single study had a disproportionate impact on the overall pooled effect, as all estimates remained within a narrow range and were statistically significant.

Residual heterogeneity remained high across iterations (I² = 63.8–86.0%), consistent with the main analysis.

These findings indicate that no single study had a disproportionate influence on the results and confirm the robustness of the pooled AUC estimate.

### Subgroup analysis

#### Major cardiac events

In the subgroup of studies specifically investigating major cardiac events [11–15], the pooled AUC was 0.82 (95% CI: 0.74–0.89), with substantial heterogeneity (I² = 76.14%, τ² = 0.0056). The prediction interval ranged from 0.65 to 0.98, suggesting variability in future estimates. Egger’s test was not significant (*z* = −0.69, *p* = *0.49*), indicating no clear evidence of publication bias.

#### Acute coronary syndromes (ACS)

Among studies focusing on acute coronary syndromes [14, 16–19], the pooled AUC was 0.81 (95% CI: 0.70–0.91), with high heterogeneity (I² = 82.7%, τ² = 0.0103). The prediction interval ranged from 0.58 to 1.03, reflecting considerable between-study variability. Egger’s test was significant (*z* = −2.41, *p* = 0.016), indicating potential publication bias.

#### CT subgroup

In the subgroup of studies using CT-based imaging (6 studies), the pooled AUC was 0.81 (95% CI: 0.72–0.90), with substantial heterogeneity (I² = 74.3%, τ² = 0.0082). The prediction interval ranged from 0.61 to 1.01, indicating variability across future studies. Egger’s test was significant (z = −5.07, *p* < 0.0001), suggesting the presence of potential publication bias.

#### MRI subgroup

In the subgroup of studies employing MRI (4 studies), the pooled AUC was 0.81 (95% CI: 0.72–0.91), again with high heterogeneity (I² = 85.5%, τ² = 0.0076). The prediction interval ranged from 0.62 to 1.01, suggesting substantial variability in effect estimates. Egger’s test was not significant (z = −0.45, *p* = 0.66), indicating no evidence of publication bias.

#### Myocardium segmentation subgroup

For studies focusing on myocardial segmentation (6 studies), the pooled AUC was 0.79 (95% CI: 0.71–0.87), with considerable heterogeneity (I² = 78.8%, τ² = 0.0072). The prediction interval ranged from 0.61 to 0.98, suggesting notable between-study variability. Egger’s test was not significant (z = −1.44, *p* = 0.15), indicating no clear evidence of publication bias.

#### EAT/PCAT segmentation subgroup

In the subgroup of studies analyzing EAT/PCAT segmentation (4 studies), the pooled AUC was 0.85 (95% CI: 0.75–0.94), with substantial heterogeneity (I² = 76.0%, τ² = 0.0067). The prediction interval ranged from 0.66 to 1.04, reflecting variability across studies. Egger’s test was significant (z = −2.87, *p* = 0.0041), suggesting possible publication bias.

#### Meta regression

To explore potential sources of heterogeneity, meta-regression analyses were performed, including continuous study-level variables that were, as far as possible, representative of potential sources of bias. Given the limited number of included studies (k = 10), only univariable models were tested to reduce the risk of overfitting and spurious associations.

When the METRICS quality score was included as a covariate, no significant association with diagnostic performance was observed (β = −0.023, 95% CI −0.70 to 0.65; *p* = 0.94), and the amount of explained heterogeneity was negligible (R² = 0%). Residual heterogeneity remained high (I² = 85.7%, *p* < 0.001).

Similarly, the number of patients included in each study was not significantly associated with the pooled AUC (β = −0.0001, 95% CI −0.0004 to 0.0002; *p* = 0.47), explaining only a small proportion of the between-study variability (R² = 4.6%).

Forest and funnel plots of subgroup analyses are shown in Supplementary Figs. S2–S7. Results are summarized in Supplementary Table S5.

## Discussion

This systematic review and diagnostic meta-analysis provide an updated, comprehensive evaluation of the current landscape of radiomics research in cardiac imaging, with a dual focus on methodological rigor—assessed using the METRICS framework—and diagnostic performance in cardiovascular risk prediction. The overall methodological quality of the studies included in this review was moderate. The average METRICS score across 202 papers was 54.5% (± 15.9%), with only 10 studies (4.9%) rated as “Excellent” and most falling into the “Good” (34.7%) or “Moderate” (40.1%) categories.

Despite a growing number of publications in recent years (32 papers in 2021 after February, 49 in 2022, 47 in 2023, 58 in 2024, and 16 in 2025 up to March 10th), there was no clear improvement in quality over time. In fact, no significant association between the score and the year of publication (Spearman’s ρ = 0.004, *p* > 0.05) was found, suggesting that the score has remained stable over time without evidence of an increasing or decreasing trend. This suggests that the increasing volume of studies has not yet been translated into stronger methodological standards.

Most studies performed well in certain crucial areas. Eligibility criteria were clearly defined in 173 out of 202 studies (85.6%), and 151 studies (74.8%) used high-quality reference standards. Core steps of radiomics workflows were consistently present: segmentation was included in 195 studies (96.5%), and hand-crafted feature extraction was reported in 194 (96.0%).

Nevertheless, several areas revealed important weaknesses. Preprocessing steps were transparently reported in 94 cases (46.5%), while clear documentation of feature extraction parameters was provided in only 79 (39.1%).

Fully automated segmentation was used in only 46 studies (22.8% of the total); a formal evaluation of the segmentation process was conducted in just 18 studies (39.1%), which is a low percentage, considering the importance of segmentation in radiomics workflows.

Additionally, a test set segmentation mask produced by a single reader or automated tool was present in 101 papers (50% of the total). This may indicate a bias in reducing heterogeneity of segmentations and, thus, of extracted radiomics features. The fact that this item is present in only 50% of papers shows that further steps are needed to increase the precision and generalizability of radiomics research in cardiovascular imaging.

Moreover, confounding factors were explicitly addressed in just 47 studies (23.3%), suggesting a frequent oversight in analytical robustness.

While internal validation was relatively common—performed in 129 studies (63.9%)—external validation appeared in only 28 cases (13.9%).

Transparency and reproducibility were critically lacking: data were made available in only 36 studies (17.8%), code in 14 (6.9%), and trained models in just 5 (2.5%). These gaps limit the possibility for independent replication and slow progress toward clinical implementation. It is interesting to note that the previous review on cardiac imaging radiomics study quality also found open science and data sharing to be a major limitation, with less than 10% of studies meeting the corresponding RQS criteria [8]. However, these limitations often reflect intrinsic barriers such as privacy regulations, ethical restrictions, and the use of small, institution-specific datasets, which make data sharing challenging. Despite these constraints, promoting transparency, methodological standardization, and code availability remains essential to enhance reproducibility and accelerate the clinical translation of radiomics research.

When analyzing methodological quality by clinical category, METRICS scores showed no statistically significant differences between groups (*p* = 0.43).

The three most represented categories are cardiomyopathies (51 papers), cardiovascular risk (31 papers), and coronary artery disease (69 papers). These groups show relatively good average METRICS scores (56.2%, 59.0%, and 54.1%, respectively). The other categories include significantly fewer papers (ranging from 2 to 23), suggesting that ischemic heart disease, plaque characterization, and cardiomyopathies remain the central topics in cardiovascular imaging, also in radiomics research.

We also decided to focus our attention on a diagnostic meta-analysis to understand the current state of research in the field of cardiovascular radiomics. To choose a topic adequate to the scope, we analyzed the number of papers and the possible outcomes: the majority of studies fall under the categories of cardiovascular risk and coronary artery disease, accounting for 100 papers—nearly half of the total. Cardiac events represent the most standardizable outcome, as they can be clearly classified as present or absent. Moreover, investigating whether imaging analysis has predictive potential is particularly relevant for guiding therapeutic decisions or follow-up strategies.

To date, no meta-analyses appear to have been conducted specifically on this topic.

From the initial pool, 17 studies were considered, and after exclusions, 10 analyses from 9 studies (totaling 2967 patients) were included. The average METRICS score among these selected studies was 63.7% (± 15.5%), notably higher than that of the overall cohort. This suggests that, at least in the field of cardiovascular event prediction, there is greater attention to research methodology.

The pooled area AUC for these studies was 0.81 (95% CI: 0.75–0.87), suggesting good diagnostic accuracy. However, substantial heterogeneity was observed (I² = 83.4%, τ² = 0.0068), and the prediction interval ranged widely from 0.64 to 0.99, indicating that future studies could produce variable results.

In Supplementary Fig. S8, we summarized the workflow of the study by Chen et al [15], which achieved the highest METRICS score among those included in the meta-analysis (total score 80.6%). This figure illustrates the main methodological steps reported in the study, serving as an example of how the METRICS framework can be applied to describe radiomics workflows.

Egger’s test was significant in the overall analysis (*p* = 0.017), suggesting potential publication bias. As demonstrated by Kocak et al [20], top clinical radiology journals almost never publish negative results, having a strong bias toward publishing positive results. In our analysis, despite not considering only Q1 journals, we observed that the results are indeed very optimistic, with some limitations to consider: only one study is prospective and uses external validation [15]; only two studies are multicentric [15, 19] and, despite using non-radiomic models in 7 out of 10 studies; no study reports a better performance of non-radiomic models than models containing radiomic variables (Table 3) and only one study reported an AUC < 0.7 [19]. Furthermore, none of the included studies freely shares the data used for the analyses.

Sensitivity and subgroup analyses confirmed the robustness of the results. The leave-one-out analysis showed pooled AUCs ranging from 0.80 to 0.83, confirming that no single study disproportionately influenced the overall results. The trim-and-fill analysis imputed three potentially missing studies on the right side of the funnel plot, yielding a bias-adjusted pooled AUC of 0.85 (95% CI: 0.79–0.91) with a prediction interval of 0.66–1.04. The adjusted estimate was nearly identical to the original, indicating that the potential effect of publication bias on the pooled diagnostic performance was minimal.

Subgroup analyses by clinical setting (MACE, acute coronary syndromes), imaging modality (CT, MRI), and segmentation target (myocardium or EAT/PCAT) revealed comparable pooled AUCs (0.79–0.85) despite substantial heterogeneity, reflecting methodological and population-related variability across studies.

Univariable meta-regression, restricted to continuous variables representative of potential bias sources (sample size and METRICS score), showed no significant associations with diagnostic performance, consistent with the small number of available studies.

Overall, these analyses support the robustness of the pooled estimates while underscoring the need for larger, prospectively designed, and methodologically homogeneous studies to validate these findings.

These results indicate that while radiomics models show promising diagnostic performance for cardiovascular risk stratification, their real-world applicability remains limited due to methodological heterogeneity and possible publication bias, as poor or inconclusive results are likely underreported. This variability underscores the need for greater standardization of imaging and analytical protocols to enhance reproducibility and generalizability. Future multicenter prospective studies with external validation are essential to confirm these findings and support the clinical translation of radiomics into cardiovascular practice.

Several limitations must be acknowledged. (1) The METRICS evaluation relies on the information reported in the publications, meaning that some studies may have been penalized for incomplete documentation despite using sound methodologies. (2) The meta-analysis focused solely on cardiovascular risk prediction, which, while methodologically consistent, limits the scope of generalizability to other cardiac conditions. (3) The number of studies included in the meta-analysis was relatively small; only nine papers and 10 studies could be included in the meta-analysis, reflecting the limited number of investigations reporting comparable quantitative data. Although this restricts the generalizability of the results, the rigorous selection ensured methodological consistency by excluding studies with heterogeneous or non-standardized endpoints. (4) Significant heterogeneity and publication bias were present, further limiting the strength of the pooled results. (5) This systematic review was not prospectively registered, and the Cochrane database was not specifically screened, which may have limited the completeness of the literature search. (6) The inter-reader agreement was not assessed for METRICS. As demonstrated by Akinci D’Antonoli et al [21], METRICS reproducibility was poor to moderate among readers both with and without training. This data must be considered when interpreting the results, which could be biased by overly optimistic or pessimistic assessments of the scores. A possible future direction could be to evaluate the reproducibility of METRICS specifically in the context of cardiovascular imaging.

In conclusion, although the mean METRICS score in our study was moderate, it should not be considered low given the heterogeneity of the included studies. Moreover, the good, pooled AUC for the prediction of myocardial ischemia and MACE, despite the presence of some publication bias, suggests that radiomics holds potential in the field of cardiovascular imaging and disease prediction. To move toward clinical translation, future studies must prioritize external validation, transparent reporting, and the sharing of data and models. Only through rigorous and open research practices can radiomics truly become a reliable component of precision cardiovascular medicine.

## Supplementary information

ELECTRONIC SUPPLEMENTARY MATERIAL

## Abbreviations

AUC: Area under the curve
EAT/PCAT: Epicardial/pericoronary adipose tissue segmentation
EuSoMII: European Society of Medical Imaging Informatics
MACE: Major adverse cardiovascular events
METRICS: METhodological RadiomICs Score
RQS: Radiomics quality score
SE: Standard error
