# Supplementary material for: CT and MRI radiomics in cardiovascular risk prediction: a systematic review and meta-analysis by the EuSoMII Radiomics Auditing Group
Source: Eur Radiol. 2025 Dec 24;36(5):4049–60. doi: 10.1007/s00330-025-12236-2 (PMC13086800; doi:10.1007/s00330-025-12236-2)
Supplement: Supplementary file 1 — ELECTRONIC SUPPLEMENTARY MATERIAL [file 330_2025_12236_MOESM1_ESM.pdf]

CT and MRI Radiomics in Cardiovascular Risk Prediction: a Systematic Review and Meta-Analysis by the EuSoMII Radiomics Auditing Group

ELECTRONIC SUPPLEMENTARY MATERIAL

Supplementary Figure S1: Funnel plot with trim-and-fill analysis.

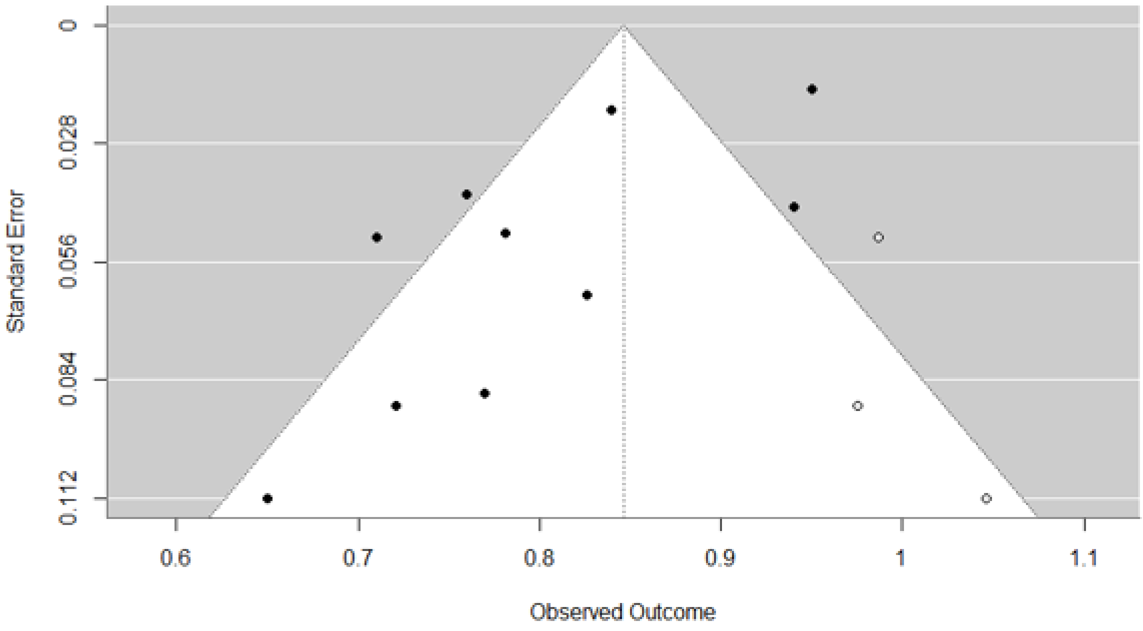

Supplementary Figure S2: Forest and Funnel plot of Subgroup of studies focused on Acute Coronary Syndrome prediction.

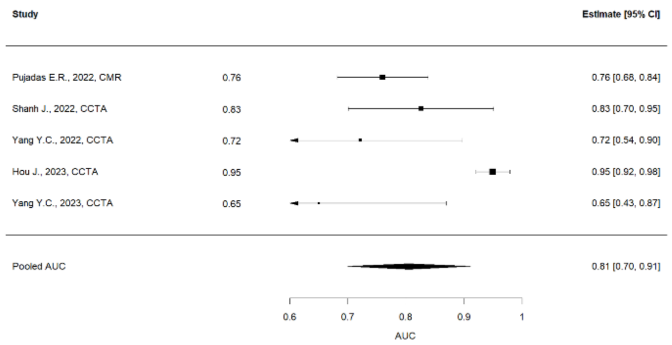

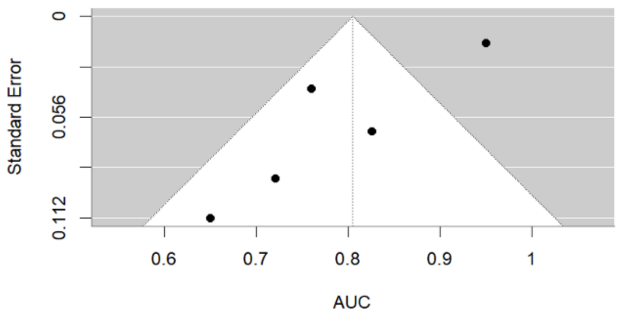

Supplementary Figure S3: Forest and Funnel plot of Subgroup of studies focused on prediction of MACE.

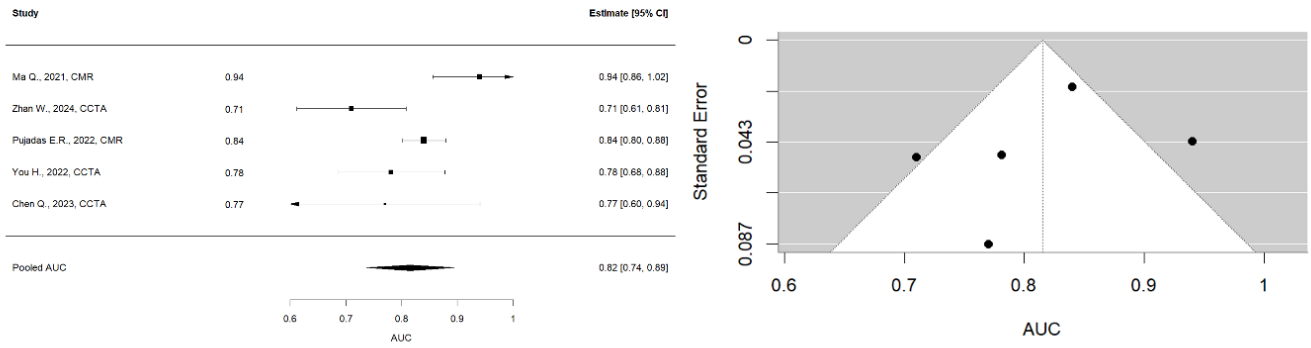

Supplementary Figure S4: Forest and Funnel plot of Subgroup of studies using CT.

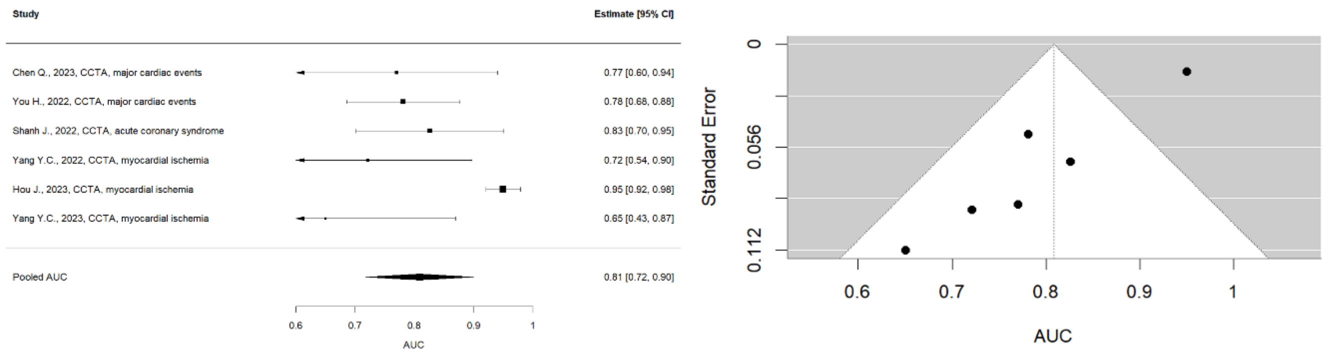

Supplementary Figure S5: Forest and Funnel plot of Subgroup of studies using MRI.

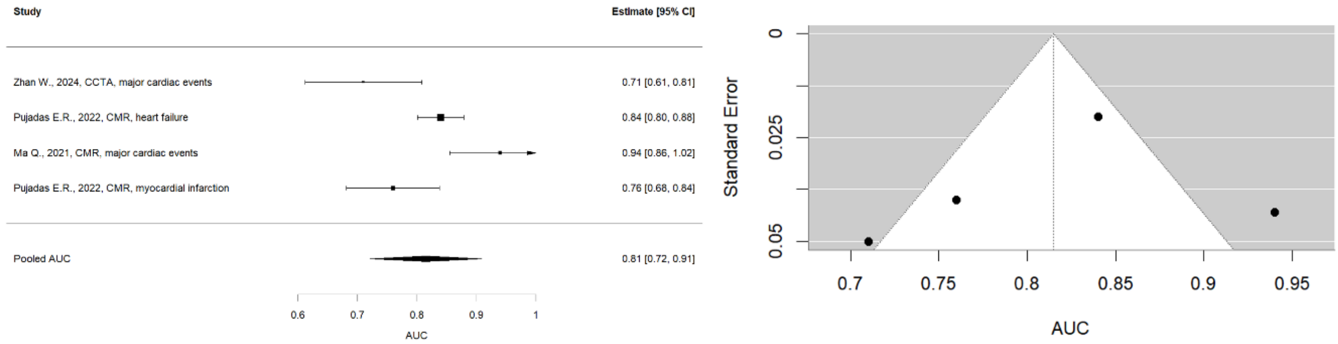

Supplementary Figure S6: Forest and Funnel plot of Subgroup of studies where myocardium was segmented.

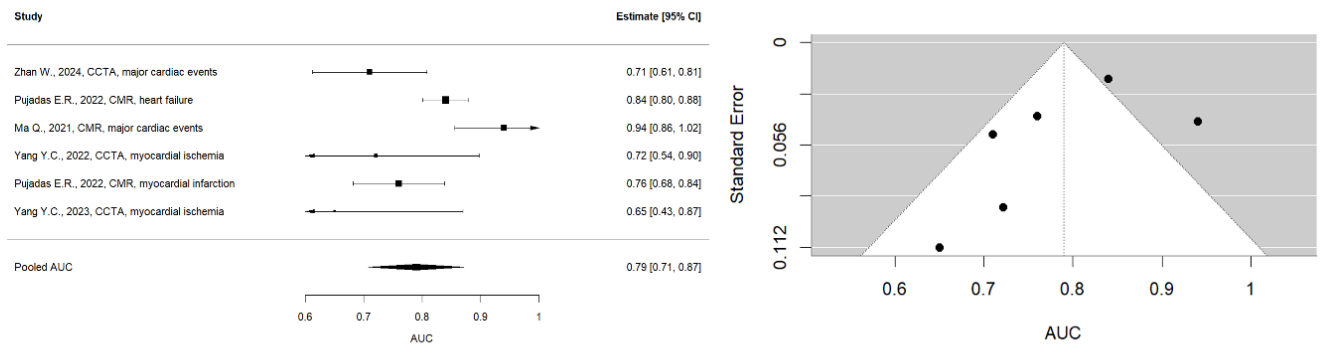

Supplementary Figure S7: Forest and Funnel plot of Subgroup of studies where EAT or PCAT was segmented.

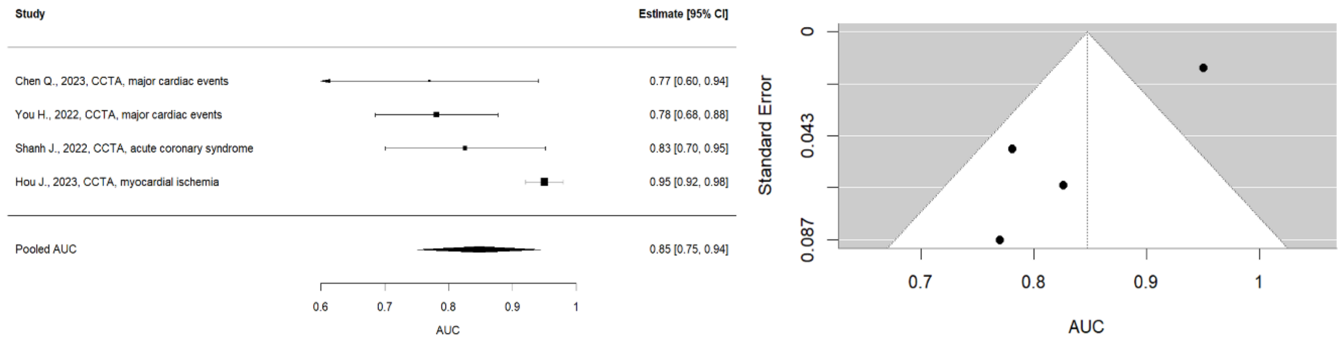

Supplementary Figure S8: Diagram showing the roadmap of the study included in the meta-analysis with the highest METRICS score: “A Coronary CT Angiography Radiomics Model to Identify Vulnerable Plaque and Predict Cardiovascular Events” by Chen Q et al [15]; total METRICS score: 80,6%. Each box represents a METRICS block; only positive items were included.

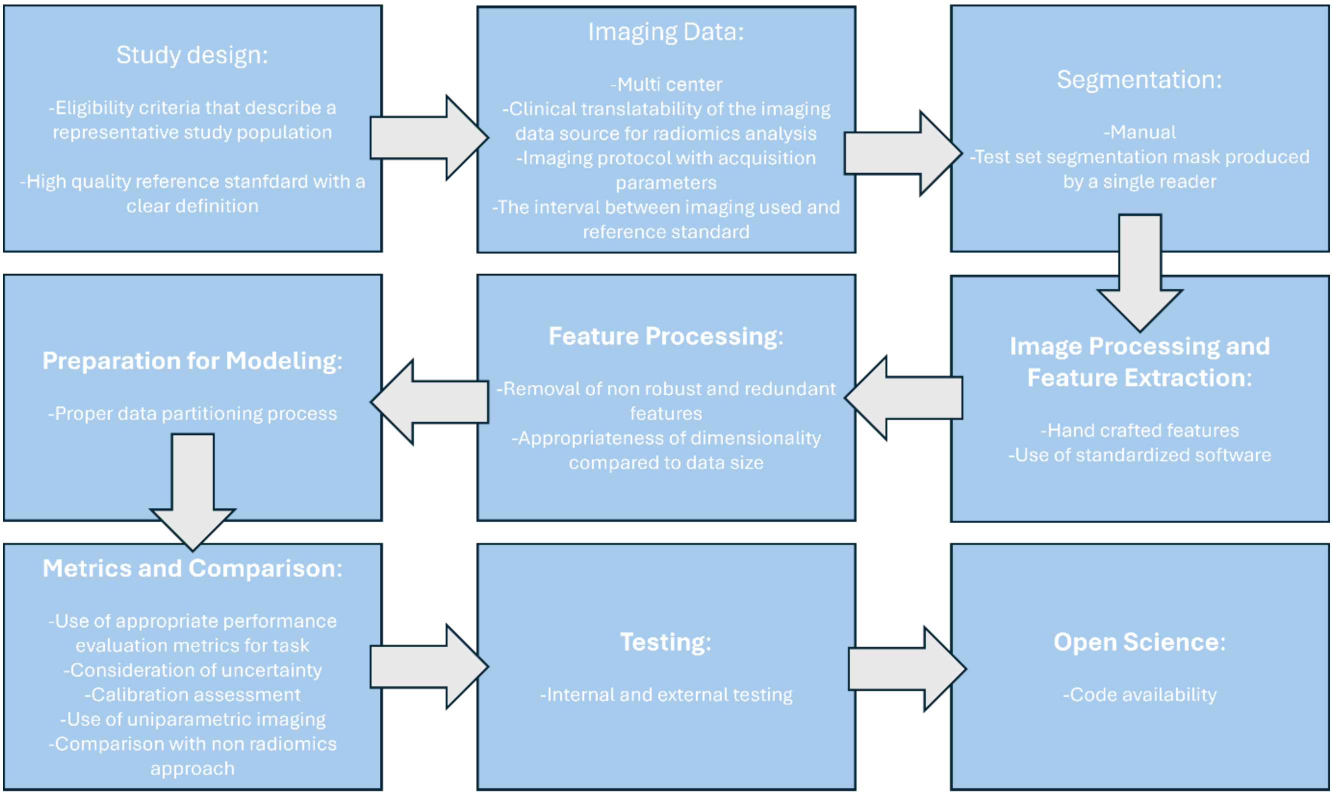

Supplementary Table S1: radiomics features selected in the included studies.

Manuscript	***Radiomic features***
Quanmei Ma et al.[11]	**Histogram**(Mean, variance, skewness, kurtosis, percentiles); **Absolute gradient** (Gradient mean, variance, skewness, kurtosis, non-zeros); **Grey-level co-occurrence matrix** (Angular second moment, contrast, correlation, sum of squares, inverse difference moment, sum average, sum variance, sum entropy, entropy,difference variance, difference entropy); **Grey-level run-length matrix** (Run-length non-uniformity, grey-level non-uniformity, long run emphasis,short run emphasis, and run percentage); **Autoregressive model** (θ1, θ2, θ3, θ4, σ); **Wavelets** (Sub-bands LL, LH, HL, and HH at five scales)
Weisheng Zhan et al [12]	N.A.
Hongrui You et al. [13]	**PCAT-score** (HighGrayLevelZoneEmphasis; Coarseness; ZoneEntropy; SmallAreaHighGrayLevelEmphasis; DependenceNonUniformityNormalized; GrayLevelVariance; InverseVariance; ClusterShade; ZonePercentage; LargeDependenceLowGrayLevelEmphasis; Small Dependence High Gray Level Emphasis; HighGrayLevelRunEmphasis); **EAT-score** (HighGrayLevelZoneEmphasis; InverseVariance)
Esmeralda Ruiz Pujadas et al. [14]	**Atrial Fibrillation**(Maximum 2D diameter slice; Energy; Maximum 2D diameter column;Maximum 2D diameter row;Dependence non-uniformity;Inverse difference moment;Large area low grey level emphasis;Large area low grey level emphasis (ES);Maximum 2D diameter row (LV ES);Surface area;Maximum 2D diameter slice (LV ED);Maximum 3D diameter;Sum of squares;Zone variance;Maximum 2D diameter row (MYO ED); Energy (LV ED);Grey level non-uniformity;Run percentage;Major axis) ; **Heart Failure**(Maximum 2D diameter slice;Minor axis;Volume (RV ED);Large area low grey level emphasis; Volume (LV ES);Informal measure of correlation;Small dependence emphasis;Grey level non-uniformity;Surface area); **Myocardial Infarction**(Coarseness;Maximum 2D diameter row (RV ED);Dependence variance;Inverse variance;Large area emphasis;Grey level variance;Sphericity (RV ES);Sphericity (MYO ED);Complexity); **Stroke**(Difference entropy; Energy; Sphericity; Joint average; Range; Large area emphasis; Sum entropy).
Qian Chen et al.[15]	original_shape_MeshVolume; wavelet.LLH_glszm_GrayLevelNonUniformity; square_gldm_GrayLevelNonUniformity;

	wavelet.HLH_glcm_MCC; wavelet.LHH_glszm_ZoneEntropy; wavelet.HHL_glcm_SumEntropy; original_shape_MajorAxisLength; wavelet.LLL_gldm_GrayLevelNonUniformity; wavelet.HLL_glszm_GrayLevelNonUniformity; wavelet.HLL_glszm_LowGrayLevelZoneEmphasis; wavelet.HHH_glcm_MCC; wavelet.LLH_gldm_SmallDependenceEmphasis; wavelet.LLH_glcm_JointEnergy; squareroot_glszm_LargeAreaHighGrayLevelEmphasis; wavelet.LHH_glcm_JointEntropy; logarithm_gldm_DependenceNonUniformity
Jin Shang et al.[16]	N.A.
You-Chang Yang et al.[17]	original_glszm_SmallAreaLowGrayLevelEmphasis; original_shape_MinorAxisLength; original_glszm_LargeAreaHighGrayLevelEmphasis; wavelet.HHH_glcm_MCC; wavelet.LHH_glszm_ZoneEntropy
Jie Hou et al.[18]	wavelet-LLL_firstorder_Mean; wavelet-LLL_firstorder_90percentile; wavelet-LLL_glcm_lmc1; log-sigma-2-0-mm-3D_firstorder_Minimum; wavelet-LLH_glrim_ShortRunLowGrayLevelEmphasis; wavelet-LLH_glcm_MaximumProbability; original _glcm_MCC
You-Chang Yang et al.[19]	original _shape_Elongation; wavelet_HLL_gidm_LargeDependenceEmphasis; log sigma 5_0 mm_30_glszm_SmallAroaEmphasis; wavelet LLL firstorder__10Percentile; log_sigma_4_0_mm_3D_giszm_SmallAreaHighGrayLevelEmphasis; wavelet_LHH_firstorder_Skerness; wavelet_LHH_g/dm_Smal:DependenceLowGrayLevelEmphasis; log_sigma_4_0_mm_3D_gidm_SmaliDependenceLowGrayLevel Emphasis

Supplementary Table S2: clinical features selected in the included studies.

Manuscript	Clinical features
Quanmei Ma et al.[11]	Age, Gender, Body mass index,Hypertension, Diabetes mellitus,Current or previous smoker, Myocardial enzymes(CK-MB; Troponin I) ;Anterior AMI, Time from symptom onset to PCI, Killip class
Weisheng Zhan et al [12]	Age,Smoking, BMI, Gender, Hypertension,Diabetes ,Baseline medications(Antiplatelet,Beta−blocker , ACEI/ARB, Statin), Lipids(Triglycerides, Total−cholesterol, LDL, HDL) Inflammatory markers(White cell count)
Hongrui You et al. [13]	Age, BMI, Gender, Hypertension, Diabetes, Hyperlipidemia, Smoking, Drinking, Lipids(CHOL,GLU, LDL-C, HDL-C, TG), Inflammatory markers(hs-CRP, Monocyte, MHR), Medications(b Blocker, Statins, Antiplatelet, CCB, ACE-I/ARB)
Esmeralda Ruiz Pujadas et al. [14]	Age, Gender, Townsend Deprivation Index,Body mass index Current smoker, Diabetes status, Hypertension status,High cholesterol status, IPAQ (MET minutes/week), Education level, Alcohol intake
Qian Chen et al.[15]	Age, Sex, BMI,Hypertension, Diabetes mellitus Hypercholesterolemia Current smoker, Clinical presentation(No chest pain,Stable angina, Typical angina, Atypical angina, Unstable Sangina, Nonanginal chest pain, Dyspnea and/or palpitation, NSTEMI) Stain therapy, Aspirin therapy
Jin Shang et al.[16]	N.A.
You-Chang Yang et al.[17]	Age, Gender,Hypertension, Hyperlipidemia, Diabetes, History of alcohol use, History of smoking
Jie Hou et al.[18]	Age, Gender, BMI,Risk factors(Diabetes,Hypertensionb, Hyperlipidaemia,Smoking in past 3 years, Alcohol in past 3 years, Interval between CCTA and MPI).
You-Chang Yang et al.[19]	Age, Gender, Hypertension, Hyperlipidemia, Diabetes, Drinking, Smoking

Supplementary Table S3: Summary of software used in the papers selected for the meta-analysis.

Study	Software(s) used	CE-FDA marked
Zhan W., 2024, CCTA, major cardiac events	Pyradiomics+SPSS	no
Chen Q., 2023, CCTA, major cardiac events	Syngo via FRONTIER	no
You H., 2022, CCTA, major cardiac events	3D slicer + A.K. Kit (GE Healthcare)	no
Pujadas E.R., 2022, CMR, heart failure	Pyradiomics+Matlab	no
Ma Q., 2021, CMR, major cardiac events	MaZda+R	no
Shanh J., 2022, CCTA, acute coronary syndrome	A.K GE Healthcare+R	no
Yang Y.C., 2022, CCTA, myocardial ischemia	CQK (CT Coronary Artery Quantitative Analysis Kit)	no
Pujadas E.R., 2022, CMR, myocardial infarction	Pyradiomics+Matlab	no
Hou J., 2023, CCTA, myocardial ischemia	CQK (CT Coronary Artery Quantitative Analysis Kit)	no
Yang Y.C., 2023, CCTA, myocardial ischemia	CQK (CT Coronary Artery Quantitative Analysis Kit)	no

Supplementary Table S4: Leave one out sensitivity analysis results.

Studio escluso	Estimate	SE	95% CI Lower	95% CI Upper	I² (%)
–1	0.7987	0.0321	0.7357	0.8616	82.0
–2	0.8275	0.0318	0.7652	0.8898	82.2
–3	0.8162	0.0348	0.7480	0.8843	85.4
–4	0.8199	0.0344	0.7525	0.8872	84.3
–5	0.8074	0.0361	0.7367	0.8782	80.4
–6	0.8161	0.0335	0.7504	0.8818	85.6
–7	0.8108	0.0347	0.7428	0.8788	86.0
–8	0.8201	0.0327	0.7561	0.8842	84.8
–9	0.7954	0.0282	0.7402	0.8507	63.8
–10	0.8230	0.0315	0.7613	0.8847	83.9

Supplementary Table S5: Summary of subgroup analyses.

Subgroup	k	Pooled AUC (95% CI)	I^2 (%)	τ^2	Prediction Interval	Egger's test (z, p)
**Major cardiac events (MACE)**	5	0.82 (0.74–0.89)	76.1	0.0056	0.65–0.98	–0.69, 0.49
**Acute coronary syndromes (ACS)**	5	0.81 (0.70–0.91)	82.7	0.0103	0.58–1.03	–2.41, 0.016
**CT**	6	0.81 (0.72–0.90)	74.3	0.0082	0.61–1.01	–5.07, <0.0001
**MRI**	4	0.81 (0.72–0.91)	85.5	0.0076	0.62–1.01	–0.45, 0.66
**Myocardium segmentation**	6	0.79 (0.71–0.87)	78.8	0.0072	0.61–0.98	–1.44, 0.15
**EAT/PCAT segmentation**	4	0.85 (0.75–0.94)	76.0	0.0067	0.66–1.04	–2.87, 0.004
